# Dimethyl­ammonium dichloridotriphenyl­stannate(IV)

**DOI:** 10.1107/S1600536812028905

**Published:** 2012-06-30

**Authors:** Yaya Sow, Libasse Diop, Gabriele Kociok-Kohn, Kieran C. Molloy

**Affiliations:** a Laboratoire de Chimie Minerale et Analytique (LACHIMIA), Departement de Chimie, Faculte des Sciences et Techniques, Universite Cheikh Anta Diop Dakar, Senegal; b Department of Chemistry, University of Bath, Bath BA2 7AY, England

## Abstract

The title salt, [(CH_3_)_2_NH_2_][Sn(C_6_H_5_)_3_Cl_2_], was obtained as a by-product of the reaction between bis­(dimethyl­ammonium) oxalate and triphenyl­tin chloride. In the stannate anion, the trigonal–bipyramidal coordination environment of the Sn^IV^ atom is defined by the phenyl groups in equatorial and the Cl atoms in axial positions. The cations are connected to adjacent anions through N—H⋯Cl and C—H⋯Cl hydrogen-bonding inter­actions, leading to a chain motif parallel to [100].

## Related literature

For background to organotin(IV) chemistry, see: Chee *et al.* (2003 ▶); Evans & Karpel (1985 ▶); Gielen *et al.* (1995 ▶); Ng & Kumar Das (1997 ▶); Zhang *et al.* (2006 ▶). For compounds containing the [Sn(C_6_H_5_)_3_Cl_2_]^−^ ion, see: Harrison *et al.* (1978 ▶); Ng (1995 ▶, 1999 ▶).
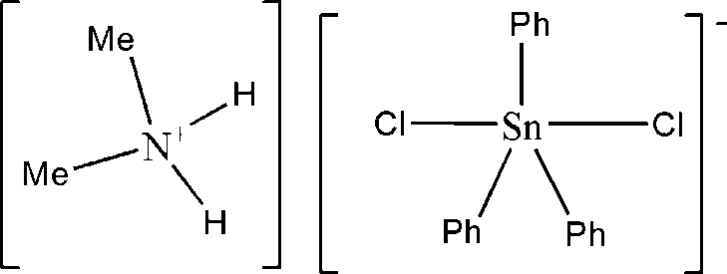


## Experimental

### 

#### Crystal data

(C_2_H_8_N)[Sn(C_6_H_5_)_3_Cl_2_]
*M* *_r_* = 466.98
Monoclinic, 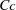

*a* = 7.9865 (1) Å
*b* = 17.5031 (3) Å
*c* = 14.9484 (3) Å
β = 105.406 (1)°
*V* = 2014.53 (6) Å^3^
*Z* = 4
Mo *K*α radiation
μ = 1.54 mm^−1^
*T* = 150 K
0.30 × 0.20 × 0.20 mm

#### Data collection

Nonius KappaCCD diffractometer
Absorption correction: multi-scan (*SORTAV*; Blessing, 1995 ▶) *T*_min_ = 0.656, *T*_max_ = 0.749
16595 measured reflections
4569 independent reflections
4469 reflections with *I* > 2σ(*I*)
*R*_int_ = 0.037

#### Refinement

*R*[*F* ^2^ > 2σ(*F* ^2^)] = 0.021
*wR*(*F* ^2^) = 0.045
*S* = 1.07
4569 reflections
227 parameters
2 restraints
H atoms treated by a mixture of independent and constrained refinement
Δρ_max_ = 0.41 e Å^−3^
Δρ_min_ = −0.89 e Å^−3^
Absolute structure: Flack (1983 ▶), 2256 Friedel pairs
Flack parameter: −0.030 (12)

### 

Data collection: *COLLECT* (Nonius, 1999 ▶); cell refinement: *DENZO* and *SCALEPACK* (Otwinowski & Minor, 1997 ▶); data reduction: *DENZO* and *SCALEPACK*; program(s) used to solve structure: *SIR97* (Altomare *et al.*, 1999 ▶); program(s) used to refine structure: *SHELXL97* (Sheldrick, 2008 ▶); molecular graphics: *ORTEP-3 for Windows* (Farrugia, 1997 ▶); software used to prepare material for publication: *publCIF* (Westrip, 2010 ▶).

## Supplementary Material

Crystal structure: contains datablock(s) I, global. DOI: 10.1107/S1600536812028905/wm2636sup1.cif

Structure factors: contains datablock(s) I. DOI: 10.1107/S1600536812028905/wm2636Isup2.hkl

Additional supplementary materials:  crystallographic information; 3D view; checkCIF report

## Figures and Tables

**Table 1:** Selected bond lengths (Å)

Sn—C7	2.152 (2)
Sn—C13	2.152 (2)
Sn—C1	2.160 (2)
Sn—Cl2	2.6098 (6)
Sn—Cl1	2.6153 (6)

**Table 2:** Hydrogen-bond geometry (Å, °)

*D*—H⋯*A*	*D*—H	H⋯*A*	*D*⋯*A*	*D*—H⋯*A*
N—H1*A*⋯Cl1	0.89 (3)	2.33 (3)	3.203 (2)	167 (3)
N—H1*B*⋯Cl2^i^	0.82 (3)	2.34 (3)	3.143 (2)	164 (3)
C2—H2⋯Cl2	0.95	2.67	3.309 (3)	125
C6—H6⋯Cl1	0.95	2.76	3.376 (2)	123
C8—H8⋯Cl1	0.95	2.70	3.344 (2)	126
C12—H12⋯Cl2	0.95	2.69	3.340 (2)	126

Symmetry code: (i) 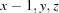.

**Fig. 1.**
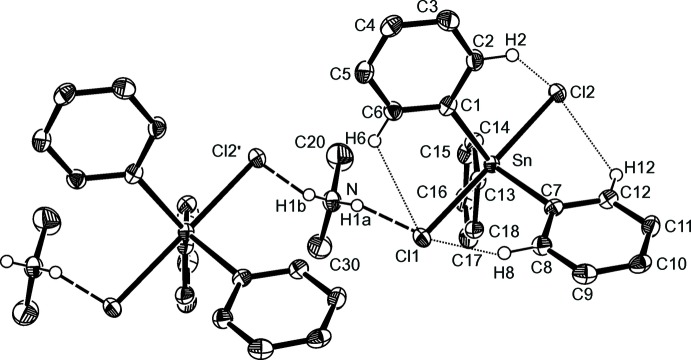
: Molecular structure of the complex showing the numbering scheme. Displacement ellipsoids are drawn at the 50% probability level.

**Fig. 2.**
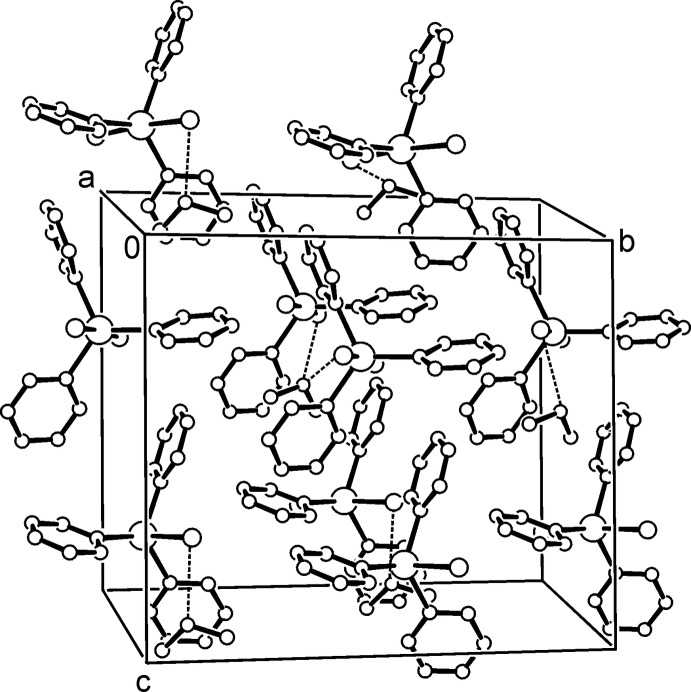
: The packing of the structure showing N—H···Cl hydrogen bonding interactions as dashed lines.

## supplementary crystallographic information

### Comment

Three [Sn(C_6_H_5_)_3_Cl_2_]^-^ stannate(IV) anion-containing compounds with 2,2'-iminodipyridinium (Ng, 1999), triphenyl(benzoylmethyl)arsonium (Harrison *et al.*, 1978) and tetramethylammonium (Ng, 1995), have previously been reported. In our research of new organotin(IV) compounds, driven by the various applications found within this family (Chee *et al.*, 2003; Evans & Karpel 1985; Gielen *et al.*, 1995; Ng *et al.*,1997; Zhang *et al.*, 2006), we have initiated here the study of the interactions between bis(dimethylammonium)oxalate and triphenyltin chloride which has yielded the title ionic product, [(CH_3_)_2_NH_2_][Sn(C_6_H_5_)_3_Cl_2_], (I).

The [Sn(C_6_H_5_)_3_Cl_2_]^-^ anion has a trigonal-bipyramidal shape with the Sn(IV) atom in a *trans*-Cl_2_C_3_ environment (Fig. 1). The equatorial plane is defined by the three phenyl groups [Sn—C 2.152 (2), 2.152 (2) and 2.160 (2) Å] while the Sn—Cl distances are 2.6098 (6) and 2.6153 (6) Å. The latter distances are very close to those reported by Ng (1995, 1999), [2.598 (1) Å] but somehow longer and shorter than those reported by Harrison *et al.* (1978) [2.573 (7), 2.689 (6) Å] for the same kind of anion. The sum of the equatorial angles (360°) indicates a planar SnPh_3_ residue, although the Cl—Sn—Cl angle deviates from linearity [174.94 (2)°].

The [SnPh_3_Cl_2_]^-^ anions are connected by the ammonium cations through a pair of similar N—H···Cl hydrogen bonds leading to an infinite chain structure parallel to [100] (Fig. 2), which is probably the origin of the Sn—Cl bond lengthening in comparison with [(CH_3_)_4_N][Sn(C_6_H_5_)_3_Cl_2_]. In the crystal packing C—H···Cl interactions are also observed (Table 1).

### Experimental

All chemicals were purchased from Aldrich (Germany) and used without any further purification. When ((CH_3_)_2_NH_2_)_2_C_2_O_4_^.^nH_2_O (obtained as a powder on submitting a 2/1 ratio mixture of [(CH_3_)_2_NH_2_][OH] and oxalic acid in water to evaporate at 333 K) is allowed to react while stirring with an excess of Sn(C_6_H_5_)_3_Cl, both as ethanolic solutions, over 2 h, a precipitate is obtained. After filtering the precipitate, slow solvent evaporation from the filtrate afforded colourless crystals of the title complex suitable for X-ray work.

### Refinement

Hydrogen atoms bonded to the N atom have been located in difference Fourier maps and have been freely refined. All other hydrogen atoms have been placed onto calculated position and refined using a riding model, with C—H distances of 0.95 Å for *sp*^2^ carbon atoms, or 0.98 Å for *sp*^3^ carbon atoms, and with *U*_iso_(H) = 1.2*U*_eq_(C) for the *sp*^2^ carbon atoms and *U*_iso_(H) = 1.5*U*_eq_(C) for the *sp*^3^ carbon atoms.

### Crystal data

**Table:**

(C_2_H_8_N)[Sn(C_6_H_5_)_3_Cl_2_]	*F*(000) = 936
*M**_r_* = 466.98	*D*_x_ = 1.540 Mg m^−^^3^
Monoclinic, *C**c*	Mo *K*α radiation, λ = 0.71073 Å
Hall symbol: C -2yc	Cell parameters from 12072 reflections
*a* = 7.9865 (1) Å	θ = 2.9–27.5°
*b* = 17.5031 (3) Å	µ = 1.54 mm^−^^1^
*c* = 14.9484 (3) Å	*T* = 150 K
β = 105.406 (1)°	Block, colourless
*V* = 2014.53 (6) Å^3^	0.30 × 0.20 × 0.20 mm
*Z* = 4	

### Data collection

**Table:**

Nonius KappaCCD diffractometer	4569 independent reflections
Radiation source: fine-focus sealed tube	4469 reflections with *I* > 2σ(*I*)
Graphite monochromator	*R*_int_ = 0.037
298 2.0 degree images with φ and ω scans	θ_max_ = 27.5°, θ_min_ = 3.5°
Absorption correction: multi-scan (*SORTAV*; Blessing, 1995)	*h* = −10→10
*T*_min_ = 0.656, *T*_max_ = 0.749	*k* = −22→22
16595 measured reflections	*l* = −19→19

### Refinement

**Table:**

Refinement on *F*^2^	Secondary atom site location: difference Fourier map
Least-squares matrix: full	Hydrogen site location: inferred from neighbouring sites
*R*[*F*^2^ > 2σ(*F*^2^)] = 0.021	H atoms treated by a mixture of independent and constrained refinement
*wR*(*F*^2^) = 0.045	*w* = 1/[σ^2^(*F*_o_^2^) + (0.0204*P*)^2^] where *P* = (*F*_o_^2^ + 2*F*_c_^2^)/3
*S* = 1.07	(Δ/σ)_max_ = 0.001
4569 reflections	Δρ_max_ = 0.41 e Å^−^^3^
227 parameters	Δρ_min_ = −0.89 e Å^−^^3^
2 restraints	Absolute structure: Flack (1983), 2256 Friedel pairs
Primary atom site location: structure-invariant direct methods	Flack parameter: −0.030 (12)

### Special details

**Table:**

Geometry. All e.s.d.'s (except the e.s.d. in the dihedral angle between two l.s. planes) are estimated using the full covariance matrix. The cell e.s.d.'s are taken into account individually in the estimation of e.s.d.'s in distances, angles and torsion angles; correlations between e.s.d.'s in cell parameters are only used when they are defined by crystal symmetry. An approximate (isotropic) treatment of cell e.s.d.'s is used for estimating e.s.d.'s involving l.s. planes.
Refinement. Refinement of *F*^2^ against ALL reflections. The weighted *R*-factor *wR* and goodness of fit *S* are based on *F*^2^, conventional *R*-factors *R* are based on *F*, with *F* set to zero for negative *F*^2^. The threshold expression of *F*^2^ > σ(*F*^2^) is used only for calculating *R*-factors(gt) *etc*. and is not relevant to the choice of reflections for refinement. *R*-factors based on *F*^2^ are statistically about twice as large as those based on *F*, and *R*- factors based on ALL data will be even larger.

### Fractional atomic coordinates and isotropic or equivalent isotropic displacement parameters (Å^2^)

**Table:**

	*x*	*y*	*z*	*U*_iso_*/*U*_eq_	
Sn	0.547277 (14)	0.045301 (7)	0.791813 (12)	0.01712 (5)	
Cl1	0.25286 (7)	0.11982 (3)	0.75104 (4)	0.02502 (13)	
Cl2	0.84601 (8)	−0.02426 (3)	0.84731 (4)	0.02268 (12)	
C1	0.4134 (3)	−0.06314 (13)	0.77445 (16)	0.0196 (5)	
C2	0.4971 (3)	−0.12794 (14)	0.75338 (18)	0.0251 (5)	
H2	0.6146	−0.1243	0.7510	0.030*	
C3	0.4122 (3)	−0.19752 (14)	0.73581 (18)	0.0293 (6)	
H3	0.4699	−0.2405	0.7191	0.035*	
C4	0.2430 (3)	−0.20458 (14)	0.74251 (19)	0.0278 (6)	
H4	0.1850	−0.2524	0.7314	0.033*	
C5	0.1592 (3)	−0.14099 (14)	0.76558 (18)	0.0269 (5)	
H5	0.0438	−0.1454	0.7711	0.032*	
C6	0.2438 (3)	−0.07124 (14)	0.78062 (17)	0.0229 (5)	
H6	0.1845	−0.0280	0.7955	0.028*	
C7	0.6122 (3)	0.08981 (12)	0.67105 (15)	0.0189 (4)	
C8	0.4818 (3)	0.11348 (13)	0.59360 (16)	0.0229 (5)	
H8	0.3639	0.1126	0.5957	0.028*	
C9	0.5229 (3)	0.13846 (14)	0.51318 (17)	0.0269 (5)	
H9	0.4331	0.1537	0.4607	0.032*	
C10	0.6947 (3)	0.14098 (13)	0.51011 (17)	0.0262 (5)	
H10	0.7225	0.1573	0.4552	0.031*	
C11	0.8259 (3)	0.11967 (13)	0.58705 (18)	0.0259 (5)	
H11	0.9440	0.1228	0.5856	0.031*	
C12	0.7843 (3)	0.09383 (13)	0.66590 (16)	0.0222 (5)	
H12	0.8750	0.0784	0.7179	0.027*	
C13	0.6162 (3)	0.10926 (12)	0.91933 (16)	0.0209 (5)	
C14	0.6982 (3)	0.07444 (14)	1.00306 (17)	0.0246 (5)	
H14	0.7210	0.0211	1.0046	0.029*	
C15	0.7473 (3)	0.11681 (15)	1.08452 (18)	0.0315 (6)	
H15	0.8030	0.0922	1.1413	0.038*	
C16	0.7159 (4)	0.19439 (15)	1.0836 (2)	0.0340 (6)	
H16	0.7496	0.2231	1.1395	0.041*	
C17	0.6349 (3)	0.23011 (15)	1.0009 (2)	0.0318 (6)	
H17	0.6148	0.2836	0.9998	0.038*	
C18	0.5825 (3)	0.18794 (13)	0.91879 (18)	0.0257 (5)	
H18	0.5240	0.2126	0.8626	0.031*	
N	0.1313 (3)	0.09715 (12)	0.93699 (16)	0.0265 (5)	
H1A	0.172 (4)	0.0954 (16)	0.887 (2)	0.034 (8)*	
H1B	0.043 (4)	0.0718 (19)	0.916 (2)	0.032 (8)*	
C30	0.0840 (5)	0.17409 (17)	0.9603 (3)	0.0535 (9)	
H30A	0.0163	0.1706	1.0061	0.080*	
H30B	0.0144	0.1993	0.9042	0.080*	
H30C	0.1897	0.2038	0.9863	0.080*	
C20	0.2435 (5)	0.0556 (2)	1.0161 (3)	0.0560 (10)	
H20A	0.3495	0.0852	1.0420	0.084*	
H20B	0.2743	0.0058	0.9952	0.084*	
H20C	0.1815	0.0482	1.0639	0.084*	

### Atomic displacement parameters (Å^2^)

**Table:**

	*U*^11^	*U*^22^	*U*^33^	*U*^12^	*U*^13^	*U*^23^
Sn	0.01594 (7)	0.01814 (7)	0.01734 (7)	−0.00084 (7)	0.00453 (5)	−0.00073 (8)
Cl1	0.0184 (3)	0.0280 (3)	0.0286 (3)	0.0049 (2)	0.0062 (2)	0.0038 (3)
Cl2	0.0170 (3)	0.0253 (3)	0.0250 (3)	0.0014 (2)	0.0043 (2)	0.0007 (3)
C1	0.0209 (11)	0.0241 (11)	0.0128 (11)	−0.0049 (10)	0.0027 (9)	−0.0002 (9)
C2	0.0238 (13)	0.0236 (12)	0.0317 (14)	−0.0029 (10)	0.0142 (11)	−0.0051 (10)
C3	0.0320 (13)	0.0230 (12)	0.0346 (15)	0.0016 (10)	0.0117 (12)	−0.0050 (11)
C4	0.0284 (13)	0.0219 (12)	0.0314 (14)	−0.0096 (11)	0.0052 (11)	−0.0040 (11)
C5	0.0205 (11)	0.0284 (12)	0.0314 (14)	−0.0053 (10)	0.0059 (10)	−0.0008 (11)
C6	0.0231 (12)	0.0239 (12)	0.0228 (13)	0.0021 (10)	0.0077 (10)	0.0005 (11)
C7	0.0223 (11)	0.0157 (10)	0.0180 (11)	−0.0031 (9)	0.0042 (9)	−0.0025 (9)
C8	0.0220 (12)	0.0231 (12)	0.0227 (13)	−0.0005 (9)	0.0043 (10)	−0.0002 (10)
C9	0.0339 (14)	0.0250 (12)	0.0200 (12)	0.0022 (10)	0.0036 (11)	−0.0004 (10)
C10	0.0422 (15)	0.0213 (12)	0.0185 (12)	0.0026 (10)	0.0136 (11)	0.0019 (10)
C11	0.0252 (13)	0.0252 (12)	0.0300 (14)	−0.0044 (10)	0.0122 (11)	−0.0017 (10)
C12	0.0230 (12)	0.0222 (11)	0.0202 (13)	−0.0020 (9)	0.0039 (10)	0.0003 (10)
C13	0.0193 (11)	0.0233 (11)	0.0207 (12)	−0.0027 (9)	0.0063 (10)	−0.0023 (10)
C14	0.0282 (13)	0.0231 (12)	0.0228 (13)	−0.0026 (10)	0.0075 (11)	−0.0014 (10)
C15	0.0358 (15)	0.0377 (15)	0.0213 (13)	−0.0058 (11)	0.0080 (12)	−0.0039 (11)
C16	0.0386 (15)	0.0391 (15)	0.0264 (15)	−0.0107 (12)	0.0127 (12)	−0.0134 (12)
C17	0.0346 (14)	0.0244 (12)	0.0406 (17)	−0.0057 (11)	0.0173 (13)	−0.0134 (12)
C18	0.0269 (13)	0.0223 (12)	0.0296 (14)	0.0001 (10)	0.0105 (11)	0.0002 (10)
N	0.0232 (11)	0.0300 (11)	0.0258 (12)	−0.0030 (9)	0.0058 (10)	−0.0033 (10)
C30	0.059 (2)	0.0319 (16)	0.081 (3)	0.0027 (14)	0.039 (2)	−0.0061 (16)
C20	0.045 (2)	0.078 (3)	0.041 (2)	0.0102 (16)	0.0024 (17)	0.0204 (17)

### Geometric parameters (Å, º)

**Table:**

Sn—C7	2.152 (2)	C11—C12	1.383 (3)
Sn—C13	2.152 (2)	C11—H11	0.9500
Sn—C1	2.160 (2)	C12—H12	0.9500
Sn—Cl2	2.6098 (6)	C13—C14	1.390 (3)
Sn—Cl1	2.6153 (6)	C13—C18	1.403 (3)
C1—C6	1.389 (3)	C14—C15	1.390 (4)
C1—C2	1.395 (3)	C14—H14	0.9500
C2—C3	1.385 (3)	C15—C16	1.380 (4)
C2—H2	0.9500	C15—H15	0.9500
C3—C4	1.387 (3)	C16—C17	1.384 (4)
C3—H3	0.9500	C16—H16	0.9500
C4—C5	1.389 (3)	C17—C18	1.397 (4)
C4—H4	0.9500	C17—H17	0.9500
C5—C6	1.385 (3)	C18—H18	0.9500
C5—H5	0.9500	N—C30	1.466 (4)
C6—H6	0.9500	N—C20	1.473 (4)
C7—C8	1.399 (3)	N—H1A	0.89 (3)
C7—C12	1.399 (3)	N—H1B	0.82 (3)
C8—C9	1.398 (3)	C30—H30A	0.9800
C8—H8	0.9500	C30—H30B	0.9800
C9—C10	1.386 (4)	C30—H30C	0.9800
C9—H9	0.9500	C20—H20A	0.9800
C10—C11	1.386 (4)	C20—H20B	0.9800
C10—H10	0.9500	C20—H20C	0.9800
			
C7—Sn—C13	119.52 (8)	C12—C11—H11	120.1
C7—Sn—C1	116.05 (8)	C10—C11—H11	120.1
C13—Sn—C1	124.43 (9)	C11—C12—C7	121.7 (2)
C7—Sn—Cl2	91.83 (6)	C11—C12—H12	119.2
C13—Sn—Cl2	87.94 (6)	C7—C12—H12	119.2
C1—Sn—Cl2	90.57 (7)	C14—C13—C18	118.7 (2)
C7—Sn—Cl1	91.49 (6)	C14—C13—Sn	121.19 (16)
C13—Sn—Cl1	87.10 (6)	C18—C13—Sn	120.14 (17)
C1—Sn—Cl1	91.40 (7)	C13—C14—C15	120.7 (2)
Cl2—Sn—Cl1	174.94 (2)	C13—C14—H14	119.6
C6—C1—C2	117.8 (2)	C15—C14—H14	119.6
C6—C1—Sn	122.82 (18)	C16—C15—C14	120.5 (3)
C2—C1—Sn	119.33 (16)	C16—C15—H15	119.7
C3—C2—C1	121.2 (2)	C14—C15—H15	119.7
C3—C2—H2	119.4	C15—C16—C17	119.6 (3)
C1—C2—H2	119.4	C15—C16—H16	120.2
C2—C3—C4	120.2 (2)	C17—C16—H16	120.2
C2—C3—H3	119.9	C16—C17—C18	120.4 (2)
C4—C3—H3	119.9	C16—C17—H17	119.8
C3—C4—C5	119.3 (2)	C18—C17—H17	119.8
C3—C4—H4	120.3	C17—C18—C13	120.1 (2)
C5—C4—H4	120.3	C17—C18—H18	120.0
C6—C5—C4	120.0 (2)	C13—C18—H18	120.0
C6—C5—H5	120.0	C30—N—C20	113.8 (3)
C4—C5—H5	120.0	C30—N—H1A	113.9 (18)
C5—C6—C1	121.4 (2)	C20—N—H1A	112.2 (19)
C5—C6—H6	119.3	C30—N—H1B	110 (2)
C1—C6—H6	119.3	C20—N—H1B	109 (2)
C8—C7—C12	117.8 (2)	H1A—N—H1B	97 (3)
C8—C7—Sn	120.62 (17)	N—C30—H30A	109.5
C12—C7—Sn	121.57 (17)	N—C30—H30B	109.5
C9—C8—C7	120.8 (2)	H30A—C30—H30B	109.5
C9—C8—H8	119.6	N—C30—H30C	109.5
C7—C8—H8	119.6	H30A—C30—H30C	109.5
C10—C9—C8	120.0 (2)	H30B—C30—H30C	109.5
C10—C9—H9	120.0	N—C20—H20A	109.5
C8—C9—H9	120.0	N—C20—H20B	109.5
C11—C10—C9	120.1 (2)	H20A—C20—H20B	109.5
C11—C10—H10	120.0	N—C20—H20C	109.5
C9—C10—H10	120.0	H20A—C20—H20C	109.5
C12—C11—C10	119.7 (2)	H20B—C20—H20C	109.5

### Hydrogen-bond geometry (Å, º)

**Table:**

*D*—H···*A*	*D*—H	H···*A*	*D*···*A*	*D*—H···*A*
N—H1*A*···Cl1	0.89 (3)	2.33 (3)	3.203 (2)	167 (3)
N—H1*B*···Cl2^i^	0.82 (3)	2.34 (3)	3.143 (2)	164 (3)
C2—H2···Cl2	0.95	2.67	3.309 (3)	125
C6—H6···Cl1	0.95	2.76	3.376 (2)	123
C8—H8···Cl1	0.95	2.70	3.344 (2)	126
C12—H12···Cl2	0.95	2.69	3.340 (2)	126

Symmetry code: (i) *x*−1, *y*, *z*.
